# Antivirulence Effects of Trans-Resveratrol and Curcumin on Methicillin-Resistant *Staphylococcus aureus* (MRSA) from Saudi Arabia

**DOI:** 10.3390/life14040491

**Published:** 2024-04-10

**Authors:** Maisa Alqahtani, May Almukainzi, Majed F. Alghoribi, Areej M. El-Mahdy

**Affiliations:** 1 Biology Department, College of Science, Princess Nourah bint Abdulrahman University, Riyadh 11671, Saudi Arabia; miss-maisa@hotmail.com; 2 Department of Pharmaceutical Sciences, College of Pharmacy, Princess Nourah bint Abdulrahman University, P.O. Box 84428, Riyadh 11671, Saudi Arabia; 3 Infectious Diseases Research Department, King Abdullah International Medical Research Center, Riyadh 11426, Saudi Arabia; alghoribima@gmail.com; 4 Department of Pathology and Laboratory Medicine, King Saud bin Abdulaziz University for Health Sciences, Riyadh 11426, Saudi Arabia; 5 Department of Pathology and Laboratory Medicine, King Abdulaziz Medical City (KAMC), Ministry of National Guard Health Affairs (MNGHA), Riyadh 11481, Saudi Arabia; 6 Department of Microbiology and Immunology, Faculty of Pharmacy, Mansoura University, Mansoura 35516, Egypt; areej@mans.edu.eg

**Keywords:** phenolic compounds, virulence factors, biofilm, hemolysin, protease, lecithinase

## Abstract

Methicillin-resistant *Staphylococcus aureus* (MRSA) is a common resistant bacterium, whose resistance has expanded to commonly used antibiotics. It is crucial to create novel treatments to tackle bacterial resistance. Trans-resveratrol and curcumin are naturally occurring phenolic compounds, whose effects on MRSA virulence are the subject of this investigation. Sub-MICs of trans-resveratrol and curcumin were tested on the virulence factors of 50 MRSA clinical isolates (CIs), including biofilm, hemolysin, hemagglutination, protease, and lecithinase. The distribution of the virulence factors of the CIs was as follows: hemolysin: 98%, hemagglutination: 70%, protease: 62%, biofilm: 56%, and lecithinase: 52%. The sub-MIC that could reduce the effect of the tested virulence factors by 50% or more (IC50) was observed in the strains that showed susceptibility to the individual administration of trans-resveratrol at 50 µg/mL and curcumin at 20 µg/mL. Hemagglutination and hemolysin activity were inhibited by at least 50% in the majority of CIs (57–94%). Meanwhile, the IC50 for protease and biofilm was observed in 6.5–17.8% of the CIs. A few of the CIs were susceptible to lecithinase inhibition, but all showed a full inhibition. This research supports the possibility of the use of these compounds to reduce the bacterial virulence that can reduce antibiotic utilization, and eventually, they can become a potential alternative treatment in combating bacterial resistance.

## 1. Introduction

Most bacterial infections in hospitals and community settings are caused by *Staphylococcus aureus*. In the USA, *S. aureus* causes more deaths than influenza, Tuberculosis (TB), viral hepatitis, and the human immunodeficiency virus combined [1]. The World Health Organization (WHO) published its first-ever list of antibiotic-resistant “priority pathogens”—a catalogue of 12 families of bacteria that pose the greatest threat to human health, of which *S. aureus* was in the second category of high priority, which contains increasingly drug-resistant bacteria [2]. Nosocomial infections have historically been associated with Methicillin-resistant *Staphylococcus aureus* (MRSA) isolates, which have rapidly developed drug resistance to multiple classes of antibiotics. The pathogen, also known as oxacillin-resistant *S. aureus* (ORSA), is currently regarded as one of the most widespread hospital-acquired infections worldwide [3].

A pathogen’s ability to infect a host is known as virulence, and “virulence factors” refer to the molecules that help the bacterium colonize the host at the cellular level, including toxins, in addition to the pathways (such as adhesion, invasion, colonization, and biofilm) that allow the pathogen to develop an infection [4]. MRSA is an infectious agent with a variety of virulence traits that can lead to diseases such as toxic shock syndrome and wound infections, food poisoning, scalded skin syndrome, and endocarditis. A number of life-threatening conditions may also occur, including pneumonia, abscesses in the brain, meningitis, and bacteremia. Virulence involves many factors and is a multifaceted process that requires coordination of a variety of components to make the organisms successful pathogens in a specific host environment. These virulence factors encourage the colonization of tissues, tissue damage, and distant illnesses [5].

The ability to combat *S. aureus* infection is provided by conventional antibiotics. Nevertheless, MRSA poses a rising risk to public health and a financial burden. Currently, vancomycin and linezolid are used as the last line of defense against MRSA to treat infections brought on by that bacterium. Vancomycin is the most recent available anti-MRSA treatment and was introduced in 1958. Daptomycin may be recommended in patients with MRSA strains that have decreased susceptibility to vancomycin [6].

Despite this, the effectiveness of these medications continues to decline as *S. aureus* resistance increases. Finding new antibiotics is getting harder, even though it is still the major strategy for treating diseases caused by bacteria. Therefore, in the battle against antibiotic-resistant *S. aureus*, it is essential to investigate alternative drug substitutes [7]. In infectious diseases, MRSA’s virulence factors are crucial, as is antibiotic resistance. Virulence factors that are implicated directly or indirectly in *S. aureus* infections include leukotoxin, hemolysin, protease, lecithinase, and biofilm. Thus, developing antimicrobials that target *S. aureus* virulence factors may be an effective strategy to treat *S. aureus*-induced infections [8]. This necessitates the creation of innovative antibacterial drugs to combat biocidal resistance [9].

Antivirulence chemicals can enhance the safety and therapeutic efficacy of antibiotics if used in combination [10]. Many polyphenolic compounds originating from plants, such as flavonoids and phenolic acids, exhibit antimicrobial action against a wide variety of pathogens and offer practical antibacterial weapons for natural warfare. Those chemicals’ therapeutic effects have led to their application in the management of human diseases. Several investigations over the past ten years have shown that combining naturally occurring plant components with widely used antibacterial medications may be a new approach to treating diseases that are brought on by bacteria that are multi-drug resistant. Plant-derived polyphenolic compounds are widely recognized to have antimicrobial effects against a variety of microbes. They are also known to sensitize drug-resistant bacterial strains to bacteriostatic or bactericidal antibiotics, making them potentially effective antimicrobial weapons in nature [11].

**Figure 1:**
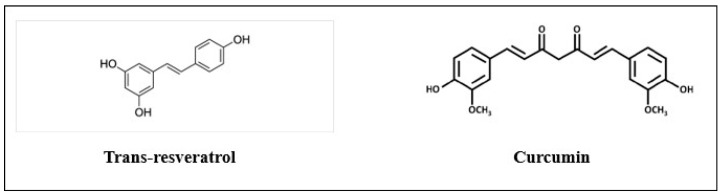
Structures of trans-resveratrol and curcumin.

Resveratrol is a stilbene polyphenol (3, 5, 4′-trihydroxy-trans-stilbene), obtained from grapes, cranberries, and peanuts, with anticancer (Figure 1), as well as antioxidant, anti-inflammatory, and estrogenic properties [12]. Previous studies have shown that it is an antivirulent compound with multiple medical benefits to human health [13]. Along with its many other bioactivities, resveratrol has the ability to prevent some harmful bacteria and fungi from growth [14]. Dose-dependent reductions in hemolytic activity and biofilm development were seen in *S. aureus* studies of different commercial red wines. Resveratrol, a major key ingredient in red wine, also prevented *S. aureus* hemolysis [15].

Turmeric (*Curcuma longa* L.) roots and rhizomes are mostly made up of curcumin, a natural polyphenol with strong antibacterial effects (Figure 1) [16]; however, numerous studies also support their inhibition of virulence factors in pathogenic bacteria. Staphylococcus aureus surface protein Sortase A (SrtA), which is implicated in bacterial adherence, was produced less frequently as a result of curcumin. Additionally, in vivo tests showed that this substance decreased bacteria’s capacity to adhere to surfaces in a dose-dependent manner [17]. Curcumin also shows many pharmacological effects, including antibacterial, antidiabetic, anti-inflammatory, anticancer, and antioxidant activities [18]. It has been demonstrated that curcumin is active against both MSSA and MRSA [19]. Curcumin decreases the development of bacterial biofilms, host receptor adhesion, and virulence factors by bacteria thanks to their quorum-sensing regulatory mechanism [20].

This study intends to investigate how curcumin and trans-resveratrol affect the virulence factors of MRSA isolates. This work advances the potential for employing natural substances that have better antibacterial properties and fewer negative effects.

## 2. Materials and Methods

### 2.1. Trans-Resveratrol and Curcumin

Trans-resveratrol was obtained from Hi-tech Development Zone, Xi’an, China. Curcumin was obtained from AK Scientific, San Francisco, CA, USA. Both compounds are 98% pure. Trans-resveratrol and curcumin stock solutions (12 mg/mL) were prepared in DMSO, while the working solutions were prepared just before each experiment. Control cultures received an equivalent amount of DMSO.

### 2.2. Isolation and Identification of Bacterial Isolates

A total of 50 clinical hospital-acquired MRSA isolates were obtained from different clinical samples, including respiratory, wound, tissue, eye swab, sterile bone, aspiration fluid, bed swab, ear, nasal swab, and groin, from two local hospitals from the cities of Riyadh and Jeddah (25 CIs from each location). Isolates were collected under medical attention with aseptic precautions from King Abdullah International Medical Research Center (KAIMARC), Kingdom of Saudi Arabia (KSA), after approval from the Institutional Review Board, Princess Nourah bint Abdulrahman University (IRB Log Number: 20-0488). VITEK 2^®^ (Biomerieux) was utilized to identify isolates and assess their susceptibility to antibiotics using standard ATCC strains of *S. aureus* (BAA977, BAA1026, BAA750, and 29213).

These MRSA isolates were screened in previous research for toxins’ genes (Staphylococcal enterotoxins *sea*, *seb*, *sec*, *sed*, and *seh*) and for panton valentine leucocidins (*lukF* and *lukS*) [21].

### 2.3. Minimum Inhibitory Concentration Assay of Trans-Resveratrol and Curcumin and Influence of Subinhibitory Concentrations on Bacterial Count

The Clinical Laboratory Standards Institute (CLSI) protocol was utilized to establish the minimum inhibitory concentration (MIC) of trans-resveratrol and curcumin against MRSA isolates [22], with some modifications as described below. Both phenolic compounds were diluted in Brain Heart Infusion (BHI) broth from a 12 (mg/mL) stock in dimethyl sulfoxide (DMSO). To obtain concentrations of trans-resveratrol and curcumin ranging from 2000 to 15.6 µg/mL, the resulting solution was thereafter 2-fold serially diluted in sterile BHI broth on a 96-well round-bottom plate. The corresponding DMSO concentrations were utilized in order to test the effect of DMSO on bacterial growth. Each concentration was generated in three parallel samples, with BHI devoid of phenolic component serving as the untreated control. MIC could be calculated as the lowest concentration at which there was no discernible bacterial growth in the broth.

Two subinhibitory concentrations of trans-resveratrol (100 and 50 µg/mL) and curcumin (50 and 20 µg/mL) were used to assess the viability of the tested isolates. Cells were serially diluted 10-fold in LB broth. The pour plate method was used to determine the count of surviving cells [23]. The number of surviving cells of treated MRSA isolates with selected concentrations of trans-resveratrol and curcumin were compared to the untreated control cells cultivated under the same conditions.

### 2.4. Effect of Trans-Resveratrol and Curcumin on Virulence Factors of MRSA

Prior to each experiment listed below, stored bacterial isolates were subcultured in LB broth at 37 °C for 18–24 h. For each isolate, three bacterial suspensions were adjusted to 0.5 MacFarland in three tubes; one of them was used as the control (untreated), and the two other tubes (treated) were treated with the chosen sub-MICs of both trans-resveratrol (50 µg/mL) and curcumin (20 µg/mL). The untreated and treated tubes were incubated overnight on a rotary shaker (150 rpm) at 37 °C. Cell-free extracts were obtained by centrifugation at 8000 rpm for 10 min at 4 °C and kept at −20 °C to be used for further evaluation of virulence factors of MRSA [24].

#### 2.4.1. Biofilm Assay

Using a polystyrene microtiter plate assay, a quantitative assessment of the biofilm production of both untreated and treated isolates with sub-MICs of both trans-resveratrol and curcumin was carried out. The biofilm was evaluated at OD492 nm [25].

#### 2.4.2. Hemolysin Assay

Trans-resveratrol and curcumin’s inhibitory effects on the release of alpha-hemolysin were evaluated using hemolysis tests [8]. Equal amounts of treated and untreated bacterial supernatants, as well as 2% RBC suspension in 10 mM Tris HCl (pH 7.4) and 160 mM NaCl, were combined and incubated in a water bath set to 37 °C for 2 h with gentle agitation to measure the hemolytic activity. By subjecting the RBCs to 0.1% sodium dodecyl sulphate (SDS) for 100% lysis and distilled water, respectively, positive and negative controls were performed. After being spun at 10.000 rpm for 10 min at 4 °C, the hemoglobin released was detected at 540 nm. In triplicate, each test was carried out independently.

#### 2.4.3. Hemagglutination Assay

The test was carried out similarly to [26]. In a U-shaped 96-well microtiter plate, 50 µL of 1% human erythrocyte suspension (O-type) and 50 µL of 2-fold serially diluted treated and untreated bacterial suspensions in PBS (readjusted to 1 McFarland) were combined. After 45 min. and 2 h of room temperature incubation, the plate was examined for agglutination.

#### 2.4.4. Total Protease Production

The skim milk assay with some modifications was used in order to assess the activity of total protease [27]. The test measures the proteolytic activity of the tested isolates by the change in the skim milk’s turbidity. Both the treated and untreated culture supernatants of the tested isolates (0.5 mL) with trans-resveratrol and curcumin were mixed with 1 mL skim milk (12.5 g/L), incubated for 30 min at 37 °C, and then, the turbidity was determined at 600 nm. Proteolytic action was carried out in triplicates.

#### 2.4.5. Assay of Lecithinase Activity

In order to assess the lecithinase activity, equal volumes of the culture supernatant with and without sub-MIC concentrations of trans-resveratrol and curcumin were combined with the egg yolk suspension. After that, this mixture was kept in an incubator overnight at 37 °C. The absorbance was determined at 600 nm. The test was run in triplicates using a 96-well microtiter plate [28].

### 2.5. Statistical Analysis

GraphPad Prism was applied to conduct the statistical analysis. A one-way ANOVA was used to evaluate the differences between the control and treated isolates. Data were represented in mean +/− standard deviation. If resveratrol and curcumin could reduce the effect of the tested virulence factors by 50% or more, this could be considered a significant effect.

## 3. Results

### 3.1. Isolation, Identification, and Antimicrobial Susceptibility Pattern of Bacterial Isolates

**Figure 2:**
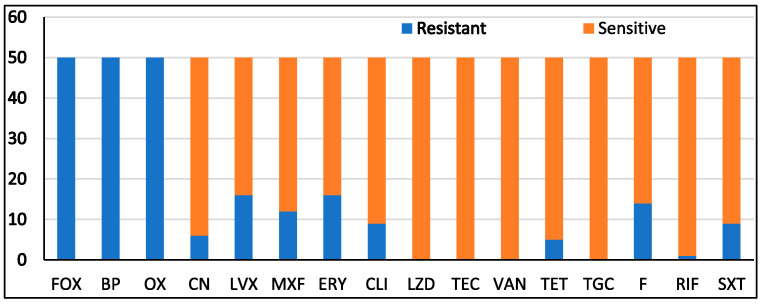
The antimicrobial susceptibility pattern of MRSA isolates.

In the current study, 50 clinical hospital-acquired isolates of MRSA, obtained from various clinical samples from Riyadh and Jeddah, were used. Thirty isolates were from wounds, five from respiratory tracts, four from tissue biopsies, four from nasal swabs, one from an eye, one from sterile bone, one from aspiration fluid, one from endo sterile, one from a bed swab, one from an ear, and one from a groin. In accordance with the standards set forth by the National Committee for Clinical Laboratory Standards, the antibiotic susceptibility patterns of the MRSA isolates were assessed using a VITEK 2 system (NCCLS, 2006). Results were interpreted as shown in (Figure 2). The results revealed resistance to cefoxitin (FOX), benzylpenicillin (BP), and oxacillin (OX) (100%); erythromycin (ERY) (34%); levofloxacin (LVX) (32%); nitrofurantoin (F) (28%); moxifloxacin (MXF) (22%); gentamicin (CN), clindamycin (CLI), and trimethoprim/sulphamethoxazole (SXT) (18%); tetracycline (TET) (10%); and rifampicin (RIF) (2%). All isolates were sensitive to linezolid (LZD), Teicoplanin (TEC), vancomycin (VAN), and tigecycline (TGC).

### 3.2. Determination of MICs of Trans-Resveratrol and Curcumin and Effect of Their Subinhibitory Concentrations on Bacterial Growth

The MICs of trans-resveratrol and curcumin were determined using the microbroth dilution method. The MIC was 500 and 125 µg/mL for all the tested isolates for trans-resveratrol and curcumin, respectively. It was noted that nearly the same bacterial count was obtained with 50 µg/mL trans-resveratrol and 20 µg/mL (160–170 × 10^6^ CFU/mL) curcumin when compared with that of the control cultures (166 × 10^6^ CFU/mL). Therefore, in further experiments, concentrations of 50 and 20 µg/mL of trans-resveratrol and curcumin, respectively, were used in the assessment of the virulence factors of MRSA isolates.

### 3.3. Detection of Virulence Factors of MRSA Isolates

**Figure 3:**
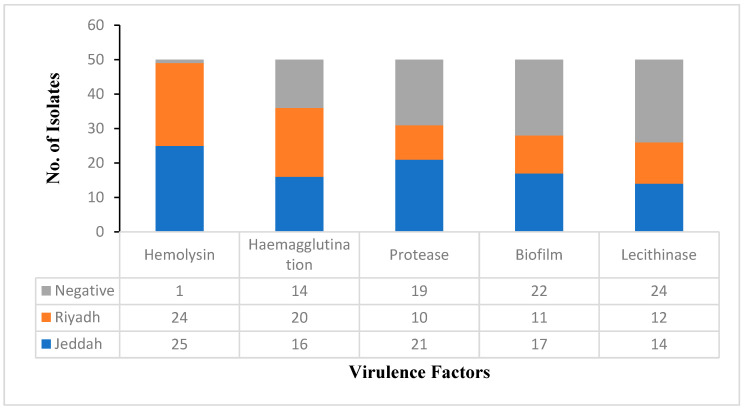
Detected virulence factors among MRSA isolates from KAIMARC, Riyadh and Jeddah, KSA.

Various virulence factors were detected in the present study using the 50 MRSA isolates, and the results indicated that the majority of isolates (98%) were hemolysin producers. This was followed by hemagglutination (70%), protease (62%), and biofilm (56%). The lowest production was observed for lecithinase, where 52% were producers. Figure 3 demonstrates the distribution of the tested virulence factors between Riyadh and Jeddah regions.

### 3.4. Virulence Profile Pattern of Studied Virulence Factors

Based on the results of the detection of virulence factors, 15 virulence patterns among the tested isolates were obtained, as shown in Table 1. Five virulence patterns were observed in Jeddah isolates only (P6, 8, 9, 13, and 14). Four patterns were found only among Riyadh isolates (P1, 11, 12, and 15). The rest of the virulence patterns were from isolates from both Riyadh and Jeddah (P2–5, P7, and P10).

### 3.5. Distribution of Detected Virulence Factors among MRSA Obtained from Several Clinical Sources

**Figure 4:**
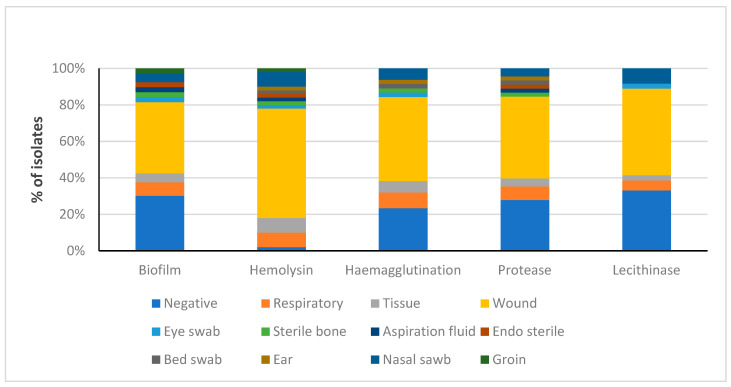
Distribution of the detected virulence factors among MRSA isolates from different clinical sources.

Figure 4 shows how different virulence factors are distributed among various isolation sources. It was found that respiratory, tissue, wound, and nasal swab isolates have all of the studied virulence factors. Wound isolates exhibited the highest prevalence of all factors.

### 3.6. Effect of Sub-MICs of Both Resveratrol and Curcumin on Virulence Factors of MRSA Isolates

#### 3.6.1. Effect on Biofilm Production

The sub-MIC concentrations of both resveratrol and curcumin significantly decreased the biofilm production in all isolates, ranging from 9 to 72%. It was noted that resveratrol and curcumin decreased the biofilm production in 14.3% and 17.8% of isolates by more than 50%, respectively, as shown in Table 2.

#### 3.6.2. Effect on Hemolysin

Results revealed that the sub-MIC concentrations of both resveratrol and curcumin decreased the hemolysin production significantly in all positive isolates (from 8 to 91%). Table 3 shows that both compounds decreased the hemolysin production in 81.6% and 57% of isolates, respectively, by more than 50%.

#### 3.6.3. Effect on Hemagglutination

Resveratrol decreased hemagglutination in 94.3% of the positive isolates, while curcumin decreased hemagglutination in 80% of the positive isolates.

#### 3.6.4. Effect on Total Protease Production

Both resveratrol and curcumin at sub-MICs significantly decreased the protease production in positive isolates. A reduction in protease of more than 50% was observed in 16% of isolates treated with resveratrol, while curcumin decreased protease by more than 50% in only 6.5% of the isolates (Table 4).

#### 3.6.5. Effect on Lecithinase Production

Both resveratrol and curcumin could decrease lecithinase production in 100% of the positive isolates, ranging from 1.3 to 28%. Neither compound could reduce lecithinase production by more than 50%, as shown in Table 5.

## 4. Discussion

Trans-resveratrol and curcumin’s effects on the virulence factors of MRSA isolates were investigated in this study. A total of 50 clinical MRSA isolates were gathered for the current investigation from various clinical sources from Riyadh and Jeddah, Saudi Arabia. The VITEK 2 system was utilized for the identification of the isolates and the determination of their patterns of antibiotic susceptibility. Figure 3 shows that most of the detected virulence factors were more prevalent in Jeddah isolates than in Riyadh isolates, except hemagglutination, which was observed more in Riyadh than in Jeddah.

The MICs of trans-resveratrol and curcumin against the tested MRSA isolates were 500 and 125 µg/mL, respectively. The findings in relation to the MIC of trans-resveratrol align with those of Duan et al. [8], who claimed that trans-resveratrol’s MIC against *S. aureus* strains was 512 µg/mL. The MIC range for resveratrol for each of the 34 strains used was 500–1000 µg/mL, as reported by Su et al. [29]. The MIC of curcumin was reported to range between 125 and 250 µg/mL in a study of 10 isolates of *S. aureus* [30]. According to Kali et al. [31], curcumin has an MIC of 126.9 g/mL against 15 Gram-positive bacteria, including 2 *Enterococcus faecalis* and 13 *S. aureus*. 

The complicated process of *S. aureus* virulence necessitates the utilization of multiple factors that must be coordinated [32]. An organism’s virulence factors will determine whether it will establish itself in the body as a pathogen or not.

A significant virulence factor is biofilm development. It encourages bacterial interaction, layering, and protection against human immunity, as well as the development of considerable multiple resistance [33]. MRSA develops a biofilm made of an extracellular polymeric matrix that is resistant to both host defense mechanisms and common antibiotics [34]. Biofilms exacerbate *S. aureus*-related skin infections [9]. This study showed that 56% of the isolates were biofilm producers. The results indicated that both phenolic compounds have the ability to eradicate biofilm. Both trans-resveratrol (50 µg/mL) and curcumin (20 µg/mL) significantly decreased biofilm production (*p* < 0.0001) by more than 50% in 14.3% and 17.8% of isolates, respectively. Inhibiting biofilms is anticipated to abolish several biofilm-associated resistance mechanisms, including delayed antibiotic penetration, persister cells, and starvation stress-mediated growth repression. Curcumin-induced membrane damage may facilitate antibiotic inflow, whereas gene expression and quorum sensing reduce bacterial pathogenicity [31]. These results coincide with those produced by Eng and Nathan [35], who reported that biofilm formation was dramatically reduced (*p* < 0.001) in the presence of 50 μM curcumin when compared to the untreated control. Pterostilbene has been shown to effectively eliminate biofilm according to Yang et al. [9]. There is a link between pterostilbene’s reduction in biofilm formation and MRSA killing in biofilms. Throughout the entire thickness of the biofilm, dead bacteria were visible. The delivery of pterostilbene may not be primarily hampered by the biofilm. Pterostilbene adhered to the biofilm surface, entered the matrix, and subsequently disseminated everywhere. The poor cell permeation of most antibacterial agents causes restricted intracellular distribution and suboptimal antibacterial effectiveness.

The effects of trans-resveratrol and curcumin on alpha-hemolysin were studied, and the results revealed that they could inhibit its production. In severe *S. aureus* infections, especially those induced by Methicillin-resistant strains, alpha-hemolysin plays a significant role in pathogenicity. The findings indicated that 98% of the isolates produced hemolysin. Regarding the effect of trans-resveratrol and curcumin on hemolysin production, it was observed that both compounds significantly reduced hemolysin production in 81.6% and 57% of isolates, respectively, by more than 50%, with a *p* value < 0.0001. Similar findings reported that trans-resveratrol inhibited *S. aureus*’ hemolytic activity [8]. The effect of trans-resveratrol may be due to reduced expressions of transcription regulator genes such as *saeR*, *saeS*, and *hla*. Previous studies described the effect of trans-resveratrol and curcumin on reducing the gene expression of different toxins, including hemolysin and panton valentine leukocidins (PVL), which is essential for the survival of pathogens in tissues for longer during infections [21]. Several studies have demonstrated how *hla*-inhibiting chemicals and medications can greatly decrease *S. aureus* virulence [36]. A study by Wang et al. [19] revealed that curcumin inhibits hla directly without affecting the growth of the organism. This might be caused by the binding of curcumin to the *Hla* “stem” region, which hindered the subsequent conformational shift of the “stem” area and inhibited the development of the heptameric transmembrane pore, hence reducing the biological activity of *Hla* for cell lysis. New and more effective antibiotics could benefit from this inhibition mechanism. 

The ability to agglutinate human erythrocytes was also assessed for all MRSA isolates. Hemagglutination was seen in 70% of the isolates. El-baz et al. [28] found similar results, reporting that hemagglutination was reported in 68.23% of isolates. The hemagglutination rates were 46.7% in Türkyilma and Kaya’s study [37], which contrasts with our findings. Trans-resveratrol decreased hemagglutination in 94.3% of the positive isolates, while curcumin decreased hemagglutination in 80% of the positive isolates. Darmani et al. [38] report that hemagglutination activity decreases in a dose-dependent manner as the curcumin concentration increases.

A bacterial protease is another virulence determinant of *S. aureus*. A number of host proteins are cleaved and degraded by staphylococcal proteases, causing tissue destruction [39]. In contrast to Saising et al. [40], who determined protease positivity in 97% of the examined isolates, 62% of our tested isolates had proteolytic activity. Some regulating factors within the host cell may contribute to the variability in protease production among clinical isolates. The used sub-MIC concentration of trans-resveratrol significantly decreased the protease production in 16% of isolates by more than 50%, while curcumin decreased protease by more than 50% in only 6.5% of the isolates, with a *p* value < 0.0001. It has also been shown that curcumin inhibits the *P. aeruginosa* PAO1 capacity to produce proteases [41]. According to El-Mahdy [12], trans-resveratrol subinhibitory doses (125 µg/mL) considerably inhibited protease synthesis in (23–53%) of the tested Gram-negative *Ps. aeruginosa* isolates.

The lecithinase enzyme has been identified in *S. aureus* associated with infections. In terms of lecithinase production, 52% of the tested MRSA isolates were lecithinase producers. Anacarso et al. [42] reported a different result, where lecithinase activity was present in just 20.5% of the clinical isolates. Our results found that both trans-resveratrol and curcumin could decrease lecithinase production in 100% of the positive isolates, ranging from 1.3 to 28%. Neither compound could reduce lecithinase production by more than 50%, as shown in Table 5, and the results were significant related to the control group, with *p* < 0.0001. These results were different from those obtained by El-Mahdy [12], who reported that trans-resveratrol at subinhibitory concentrations greatly increased the lecithinase activity in the Gram-negative *Ps. aeruginosa* isolates.

It was reported that both compounds were of poor solubility and bioavailability if used orally, and this limits their use for a long time. A recently developed strategy that could accelerate the solubility and bioavailability of both polyphenols is the use of nanoformulations of resveratrol [43] and curcumin [44], which could be used as drug delivery method for the treatment of various infections.

## 5. Conclusions

This research sought to assess the possible use of naturally occurring phenolic chemicals such as trans-resveratrol and curcumin as effective alternatives for antimicrobial agents through a reduction in the bacterial virulence of drug-resistant bacteria. Both compounds, in their subinhibitory concentrations, could decrease the studied virulence factors of the tested MRSA isolates without affecting bacterial growth. It can be concluded that trans-resveratrol and curcumin have the potential to be effective in the treatment of bacterial infections through reducing their virulence. Also, they may be used in combination with other antibiotics to boost their antimicrobial activity through their effect on bacterial virulence.

## Figures and Tables

**Table 1:** Virulence profile patterns among tested MRSA isolates.

Virulence Profile Pattern	Virulence Factors	VFs	Riyadh	Jeddah	Total
P1	Bio, He, HA, Pro, Li	5	0	5	5
P2	Bio, He, HA, Li	4	3	1	4
P3	Bio, He, Pro, Li	4	3	2	5
P4	He, HA, Pro, Li	4	2	3	5
P5	Bio, He, HA, Pro	4	1	4	5
P6	HA, Pro, Li	3	1	0	1
P7	He, HA, Pro	3	2	3	5
P8	Bio, He, HA	3	2	0	2
P9	He, HA, Li	3	3	0	3
P10	Bio, He, Pro	3	1	3	4
P11	He, Pro, Li	3	0	1	1
P12	Bio, He, Li	3	0	2	2
P13	He, HA	2	6	0	6
P14	Bio, He	2	1	0	1
P15	He, Li	2	0	1	1

VFs: virulence factors. P: pattern. Bio: biofilm, He: hemolysin, HA: hemagglutination, Pro: protease, Li: Lecithinase.

**Table 2:** Effect of trans-resveratrol and curcumin on biofilm reduction by more than 50% of biofilm-positive isolates.

Isolate No.	Control	Trans-Resveratrol	% Reduction	Curcumin	% Reduction
17	0.14 +/− 0.017	0.07 +/− 0.002	50.7	-	-
29	0.34 +/− 0.001	0.11 +/− 0.001	66.8	0.11 +/− 0.001	68.2
36	0.21 +/− 0.005	-	-	0.10 +/− 0.001	50.1
40	0.22 +/− 0.001	0.08 +/− 0.002	65	0.10 +/− 0.002	54.7
50	0.29 +/− 0.001	0.08 +/− 0.001	70.7	0.07 +/− 0.001	72.6

Data presented as mean +/− SD, *n* = 3.

**Table 3:** Hemoglobin release reduction by more than 50% in trans-resveratrol- and curcumin-treated positive samples.

Isolate No.	% Hemoglobin Release				
	Control	Trans-Resveratrol	% Reduction	Curcumin	% Reduction
2	53.85 +/− 0.031	7.05 +/− 0.065	87	-	-
4	24.07 +/− 0.035	11.84 +/− 0.046	50.8	6.09 +/− 0.014	74.7
5	7.07 +/− 0.025	3.24 +/− 0.072	54.2	-	-
6	5.76 +/− 0.02	2.24 +/− 0.025	61.2	-	-
7	13.14 +/− 0.06	3.21 +/− 0.021	75.6	3.20 +/− 0.025	75.6
9	21.17 +/− 0.025	3.81 +/− 0.026	82	5.14 +/− 0.021	75.7
10	9.94 +/− 0.032	2.55 +/− 0.031	74.3	3.22 +/− 0.017	67.6
11	9.66 +/− 0.119	3.81 +/− 0.026	60.6	3.52 +/− 0.015	63.5
14	3.82 +/− 0.025	1.25 +/− 0.031	67.2	-	-
15	21.47 +/− 0.015	3.52 +/− 0.015	83.6	2.88 +/− 0.01	86.6
17	3.52 +/− 0.012	1.52 +/− 0.02	56.8	1.65 +/− 0.02	53.2
18	9.26 +/− 0.061	-	-	3.21 +/− 0.01	65.3
19	21.78 +/− 0.021	3.20 +/− 0.015	85.3	4.81 +/− 0.015	78
20	13.47 +/− 0.031	3.53 +/− 0.021	73.8	4.22 +/− 0.072	68.7
21	6.73 +/− 0.040	2.56 +/− 0.006	62	-	-
22	8.04 +/− 0.074	3.83 +/− 0.010	52.3	3.19 +/− 0.010	60.3
23	13.48 +/− 0.020	3.19 +/− 0.015	76.3	4.80 +/− 0.010	64.4
24	13.10 +/− 0.096	2.87 +/− 0.006	78	4.16 +/− 0.006	68.2
25	50.00 +/− 0.10	9.29 +/− 0.010	81.4	-	-
27	27.55 +/− 0.021	4.18 +/− 0.020	84.8	5.76 +/− 0.006	79
28	3.20 +/− 0.015	1.12 +/− 0.012	64.8	1.41 +/− 0.017	56
29	77.88 +/− 0.02	15.70 +/− 0.015	79.8	35.24 +/− 0.012	54.7
31	87.56 +/− 0.122	15.70 +/− 0.010	82	-	-
32	81.74 +/− 0.010	12.81 +/− 0.010	84.3	-	-
33	27.26 +/− 0.032	7.05 +/− 0.050	74	13.22 +/− 0.020	51.5
34	14.73 +/− 0.010	5.43 +/− 0.012	63	-	-
35	34.60 +/− 0.010	6.41 +/− 0.006	81.5	5.11 +/− 0.015	85.2
36	61.53 +/− 0.006	9.91 +/− 0.015	84	19.67 +/− 0.058	68
37	85.89 +/− 0.010	12.49 +/− 0.006	85.5	24.37 +/− 0.025	71.6
38	21.15 +/− 0.010	8.99 +/− 0.015	57.5	-	=
39	76.29 +/− 0.010	6.41 +/− 0.020	91.6	10.58 +/− 0.015	86
40	81.42 +/− 0.006	23.69 +/− 0.015	71	-	-
41	68.27 +/− 0.026	17.62 +/− 0.006	74.2	-	-
42	89.73 +/− 0.010	21.09 +/− 0.081	76.5	25.35 +/− 0.132	71.7
44	3.20 +/− 0.010	1.13 +/− 0.144	64.6	0.97 +/− 0.010	69.7
45	4.18 +/− 0.020	1.78 +/− 0.015	57.3	2.07 +/− 0.026	50.5
46	15.38 +/− 0.015	4.16 +/− 0.010	73	3.54 +/− 0.015	77
48	17.94 +/− 0.006	5.76 +/− 0.010	68	3.83 +/− 0.006	78.6
49	20.86 +/− 0.038	4.80 +/− 0.010	77	6.73 +/− 0.010	67.7
50	99.32 +/− 0.015	20.33 +/− 0.101	79.5	-	-

Data presented as mean +/− SD, *n* = 3.

**Table 4:** Effect of trans-resveratrol and curcumin on protease production in protease-positive isolates relative to the negative control.

Isolate No.	Protease Production		
	Control	Trans-Resveratrol	Curcumin
33	67.97 +/− 0.021	21.27 +/− 0.007	-
34	60.14 +/− 0.015	28.44 +/− 0.015	-
36	49.28 +/− 0.006	24.35 +/− 0.010	25.27 +/− 0.002
38	50.39 +/− 0.015	17.96 +/− 0.058	7.65 +/− 0.001
42	60.18 +/− 0.015	29.45 +/− 0.010	-

Data presented as mean +/− SD, *n* = 3.

**Table 5:** Effect of trans-resveratrol and curcumin on lecithinase production in lecithinase-positive isolates.

Isolate No.	Lecithinase Production				
	Control	Trans-Resveratrol	% Reduction	Curcumin	% Reduction
1	1.37 +/− 0.0006	1.18 +/− 0.006	14.2	1.0.6 +/− 0.001	22.7
9	1.35 +/− 0.0006	1.28 +/− 0.001	4.9	1.24 +/− 0.001	8.5
11	1.27 +/− 0.001	1.13 +/− 0.001	11.5	0.99 +/− 0.001	22
13	1.53 +/− 0.0006	1.51 +/− 0.001	1.3	1.41 +/− 0.002	7.9
14	1.55 +/− 0.0006	1.40 +/− 0.01	9.7	1.43 +/− 0.02	7.7
16	1.88 +/− 0.0006	1.83 +/− 0.006	2.4	1.63 +/− 0.001	13.3
17	1.87 +/− 0.0006	1.49 +/− 0.001	19.7	1.38 +/− 0.001	25.9
21	1.82 +/− 0.002	1.78 +/− 0.001	2.6	1.70 +/− 0.001	6.5
22	1.85 +/− 0.002	1.79 +/− 0.002	3.02	1.65 +/− 0.002	10.6
23	1.87 +/− 0.0006	1.72 +/− 0.002	7.76	1.69 +/− 0.006	9.1
24	1.63 +/− 0.015	1.47 +/− 0.001	10	1.48 +/− 0.001	9.2
25	2.18 +/− 0.0006	1.85 +/− 0.002	15.2	1.75 +/− 0.001	19.8
28	2.03 +/− 0.0006	1.89 +/− 0.002	2.4	1.81 +/− 0.002	10.5
30	2.06 +/− 0.012	1.79 +/− 0.001	13	1.77 +/− 0.001	14
34	2.21 +/− 0.001	1.84 +/− 0.001	17.2	1.98 +/− 0.002	10.7
35	2.17 +/− 0.001	1.95 +/− 0.001	10.2	1.87 +/− 0.002	13.7
36	2.21 +/− 0.002	2.07 +/− 0.002	6.1	2.12 +/− 0.002	3.7
37	2.17+/− 0.0006	2.10 +/− 0.003	3	2.07 +/− 0.001	4.4
39	1.99 +/− 0.003	1.81 +/− 0.001	9.6	1.62 +/− 0.001	19
40	2.03 +/− 0.001	1.89 +/− 0.001	6.8	1.96 +/− 0.010	3.4
41	2.38 +/− 0.015	2.1 +/− 0.002	11.5	2.04 +/− 0.051	14
43	2.16 +/− 0.015	1.97 +/− 0.002	8.5	1.84 +/− 0.001	14.9
44	1.92 +/− 0.001	1.79 +/− 0.002	6.4	1.79 +/− 0.002	6.2
47	1.92 +/− 0.001	1.73 +/− 0.001	9.6	1.4 +/− 0.001	27
48	2.26 +/− 0.002	1.67 +/− 0.001	26	1.66 +/− 0.002	26.4
50	1.9 +/− 0.002	1.35 +/− 0.002	28.8	1.69 +/− 0.002	10.9

Data presented as mean +/− SD, *n* = 3.

## Data Availability

Data from this work are available from the corresponding author on request.
